# Users’ satisfaction levels about mHealth applications in post-Covid-19 times in Saudi Arabia

**DOI:** 10.1371/journal.pone.0267002

**Published:** 2022-05-04

**Authors:** Turki M. Alanzi

**Affiliations:** Health Information Management and Technology Department, College of Public Health, Imam Abdulrahman Bin Faisal University, Dammam, Saudi Arabia; Universita degli Studi della Campania Luigi Vanvitelli, ITALY

## Abstract

**Purpose:**

This study aims to investigate the users’ satisfaction levels about mHealth applications and their intentions to use them in future (in post-Covid-19 times) in Saudi Arabia.

**Methods:**

A cross-sectional survey design was adopted in this study. The mHealth App Usability Questionnaire (MAUQ)22 was used in this study. An online version of the survey was created using Google Surveys, and a link for the survey was generated. As the objective of this study is to assess the users’ satisfaction levels about mHealth applications and their intentions to use them in future (in post-Covid-19 times) in Saudi Arabia, all individuals who used or using mHealth applications will be included in this study. At the end of the survey time period, 318 responses were received.

**Results:**

Items including ’easy to learn using the app’ (mean rating = 3.9), ’easy to find information on the application’(mean rating = 3.9), ’interface of the app’ (mean rating = 3.8) were rated to be highly effective by the participants.). However, ability to recover from mistakes while using the applications (mean rating = 2.9), inconsistency in navigation (mean rating = 2.9), and lack of all necessary functions (mean rating = 2.3) were few issues identified. No statistically significant difference of opinions was observed in relation to all sub-scales of usability factor.

**Conclusion:**

Although the satisfaction levels are good with respect to mHealth applications, the sudden rise in the mHealth users can be partly linked to the several psychological issues such as anxiety and depression among people and also because of restricted access during the pandemic.

## Introduction

Since December 2019, the Covid-19 pandemic has emerged which led to the development of various variants, leading to series of lockdowns, curfews, closure of schools, businesses and other institutions, as a result of which, people in different nations started to use mHealth applications for their healthcare needs (as the healthcare systems were already strained due to Covid-19 patients). As of 13th February 2022, there are 404.9 million confirmed cases and 5.7 million deaths, reflecting the seriousness of the pandemic [1]. Different countries are experiencing different number of waves with the emergence of new variants of the novel coronavirus [2]. For instance, Saudi Arabia, one of the fastest developing countries in the Middle East has experienced two waves of infections in 2020 and 2021 [3], during which various preventive methods such as lockdowns, curfews, and closure of businesses and educational institutions were implemented.

Considering these restrictions, it was highly necessary for the people to rely on the remote healthcare technologies such as mHealth, Telehealth etc. Furthermore, healthcare transformation programme as a part of vision 2030 which included rapid digitalization of healthcare system has proved to be beneficial in delivering the healthcare services as there was a significant increase in the deployment of various mHealth application and also increase in the number of mHealth users [4]. However, studies [5–8] have identified issues in mHealth implementation in Saudi Arabia such as lack of awareness of mHealth among the citizens, and lack of facilitating conditions. In addition, varying levels of accessibility, utilization, and perceptions of digital health technologies were identified across different population groups in Saudi Arabia [9].

Several interesting aspects were identified with respect to the acceptance of eHealth applications among the Saudi Arabian users. Among the influential factors, perceived usefulness and ease of use were identified to be the major factors affecting the attitudes of the mHealth users [10]. Insufficient skills and competencies, lack of willingness to use mHealth technologies among the physicians was also identified [11]. Furthermore, issues on cloud platforms, data security, privacy, and data transfer issues are some serious problems affecting the use of mHealth applications. In addition, mediating variables such as age, gender, residence, income, education and culture have variable impact on the adoption of mHealth applications [12–16]. Furthermore, mHealth market is expected to grow at a significant rate in the next few years. In 2021, mHealth global market size was identified to be $99 billion, and is expected to reach $332.7 billion in 2025 [17]. Furthermore, mHealth applications are being developed in various health segments, including health, productivity, sports, behavioral therapy, nutrition etc., which has significantly increased the scope for adoption of mHealth applications [18]. Similarly, Ministry of Health, Saudi Arabia has launched various mHealth applications such as Mawid andSehha,. for various purposes including appointment booking, online consultations, medicine delivery etc. during the Covid-19 pandemic, which led to the significant rise in the number of mHealth users in the country [19, 20]. However, the reason for rapid rise in the number of mHealth users was not users’ interest in adoption, but necessity. Accordingly, a recent study [21] has identified that fear, stress, and depression factors among the people during the pandemic rather than self-interest, usefulness, enjoyment, ease of use etc. has led to the rapid increase in mHealth adoption.

Therefore, there is an ambiguity surrounding the rise in number of mHealth users, whether the change is led by the digitization approach of Vision 2030 in healthcare or increase in skills and awareness of the people relating mHealth technologies or the people adopted mHealth applications only to receive healthcare services during the pandemic as the access to the regular healthcare services was significantly affected. Therefore, it is important to assess if the mHealth users are satisfied or not and if they would continue to use the mHealth application in the post-Covid-19 times, as the restrictions were lifted and access to the care has improved. In this context, this study aims to investigate the users’ satisfaction levels about mHealth applications and their intentions to use them in future (in post-Covid-19 times) in Saudi Arabia.

## Material and methods

A cross-sectional survey design was adopted in this study for collecting the data related to the users’ satisfaction levels of mHealth applications in the post-Covid-19 times.

### Questionnaire design

The questionnaire focuses on various aspects of users’ satisfaction, and also other influential factors. The mHealth App Usability Questionnaire (MAUQ) [22] was used in this study. The survey questionnaire has two parts. The first part includes eight questions about participants’ demographic information. The second part focuses on the 24 main survey questions. The second part includes three sections: ease of use and satisfaction (eight items), system information arrangement (six items), and usefulness (seven items). An additional section, ’intention to use’ is added by the authors which has three items that focus on assessing the users’ intention to use mHealth applications in future. All the survey questions are answered using Likert scale ratings (on a scale of five).

The questionnaire was translated into Arabic using two professional translators. To check the reliability of the questionnaire, a pilot study was conducted with 16 randomly selected mHealth users. The results achieved Cronbach’s alpha greater than 0.70 for all the items, reflecting good internal reliability and consistency [23]. An online version of the survey was created using Google Surveys, and a link for the survey was generated.

### Sampling and participants

As the objective of this study is to assess the users’ satisfaction levels about mHealth applications and their intentions to use them in future (in post-Covid-19 times) in Saudi Arabia, all individuals who used or using mHealth applications will be included in this study. Considering the total 35 million population in Saudi Arabia [24], estimated sample was calculated using Cochran’s formula [25], at 95% CI and 5% of Margin of error, giving an estimated sample of 385 participants. The survey link was active from 10^th^ December 2021 to 9^th^ February 2022, and at the end of the survey time period, 318 responses were received. After removing the incomplete responses, 301 responses were considered for the data analysis.

### Data analysis

Data was analyzed using SPSS version 20.0, and various statistical techniques including t-tests, means, and standard deviations were used. Missing data was removed in order to avoid any bias in analyzing the results.

### Ethical considerations

Informed consent was taken from all the participants using checkbox option before starting the survey, where the users are provided with the information about the study. Anonymity of the participants is ensured in this study. Ethical approval for the study was received from Imam Abdulrahman Bin Faisal University, Saudi Arabia.

## Results

The findings revealed appropriate representation from all groups of participants, including gender, education level, age, employment status, marital status, and place of living. Focusing on gender characteristics, 49.5% of the total participants were males and 50.5% were females. Majority of the participants had good educational levels, represented by bachelor’s degree (38.5%), master’s degree (13.6%), and doctorate (4%). Whereas, 23.6% of the total participants had diploma as their education level, followed by 15.6% having high school education, and 4.7% having other qualifications. Majority of the participants included younger participants representing 20.3% from the age group of 18–29 years and 44.2% from the age group of 30–39 years. Whereas, 25.6% belonged to 40–49 years age group, 7% belonged to 50–59 years age group, and 3% participants were aged more than 59 years. In relation to the employment status, 61.1% were employed while 38.9% were unemployed or retired. Majority of the participants were married, represented by 71.4% of the total participants. Moreover, 61.1% of the total participants reside in urban areas, while 38.9% in rural areas. In relation to the mobile phone usage, majority of the participants were using mobile phones for more than ten years (47.5%), followed by 25.9% using mobile phones for seven to nine years, 22.6% for four to six years, and four percent for one to three years (Table 1). However, in relation to the use of mHealth applications, majority of the participants started using them in the last four years (50.5%), followed by 19.3% participants using for four to six years, and 30.2% using them in the last one year.

**Table 1:** Participants’ demographic information.

Demographic characteristics	N	Relative frequency
**Gender**		
Male	149	49.5%
Female	152	50.5%
**Education**		
High school	47	15.6%
Diploma	71	23.6%
Bachelor’s degree	116	38.5%
Master’s degree	41	13.6%
Doctorate	12	4.0%
Others	14	4.7%
**Age (years)**		
18–29	61	20.3%
30–39	133	44.2%
40–49	77	25.6%
50–59	21	7.0%
>59	9	3.0%
**Employment status**		
Employed	187	61.1%
Unemployed/Retired	114	38.9%
**Marital Status**		
Single	86	28.6%
Married	215	71.4%
**Place of living**		
Rural	117	38.9%
Urban	184	61.1%
**Years of using mobile devices**		
<1 year	0	0.0%
1–3 years	12	4.0%
4–6 years	68	22.6%
7–9 years	78	25.9%
10 or more years	143	47.5%
**Years of using mHealth applications**		
<1 year	91	30.2%
1–3 years	152	50.5%
4–6 years	58	19.3%
7–9 years	0	0.0%
10 or more years	0	0.0%

Focusing on the findings related to ease of use and satisfaction of mHealth applications (as presented in Table 2) among the participants, items including ’easy to learn using the app’ (mean rating = 3.9), ’easy to find information on the application’(mean rating = 3.9), ’interface of the app’ (mean rating = 3.8) were rated to be highly effective by the participants. In relation to the ’overall satisfaction’ (mean rating = 3.8), majority of the participants stated that they were satisfied. However, few issues relating to the mHealth applications, especially ’ease of use’, ’comfort in using social settings’, ’time involved in using the applications’ were rated to be slightly low compared to other items.

**Table 2:** Users perceptions of ease of use and satisfaction.

Items	Mean Rating
The app was easy to use.	3.2
It was easy for me to learn to use the app.	3.9
I like the interface of the app.	3.8
The information in the app was well organized, so I could easily find the information I needed.	3.9
I feel comfortable using this app in social settings.	3.1
The amount of time involved in using this app has been fitting for me.	3.1
I would use this app again.	3.6
Overall, I am satisfied with this app.	3.8

Focusing on the findings related to system information and arrangement of mHealth applications (as presented in Table 3), the participants acknowledged the good interface for using all functions (mean rating = 3.8) and acceptable way to receive services (mean rating = 3.9). However, ability to recover from mistakes while using the applications (mean rating = 2.9), inconsistency in navigation (mean rating = 2.9), and lack of all necessary functions (mean rating = 2.3) were few issues identified.

**Table 3:** Users perceptions of system information and arrangement.

Items	Mean Rating
Whenever I made a mistake using the app, I could recover easily and quickly.	2.9
This mHealth app provided an acceptable way to receive health care services.	3.9
The app adequately acknowledged and provided information to let me know the progress of my action.	3.4
The navigation was consistent when moving between screens.	2.9
The interface of the app allowed me to use all the functions (such as entering information, responding to reminders, viewing information) offered by the app.	3.8
This app has all the functions and capabilities I expect it to have.	2.3

Focusing on the findings related to the usefulness of mHealth applications (as presented in Table 4), all the items were rated to be effective by the participants. The apps were useful for health and well-being (mean rating = 3.9), as they had good opportunity to interact with healthcare providers (mean rating = 3.9). Furthermore, the apps were useful for managing their health effectively (mean rating = 3.8). Although the apps made it easy to communicate with healthcare providers (mean rating = 3.7), the participants were not comfortable in interacting with the healthcare providers using the applications (mean rating = 2.6).

**Table 4:** Users perceptions of usefulness.

Items	Mean Rating
The app would be useful for my health and well-being.	3.9
The app improved my access to health care services.	3.6
The app helped me manage my health effectively.	3.8
The app made it convenient for me to communicate with my health care provider.	3.7
Using the app, I had many more opportunities to interact with my health care provider.	3.9
I felt confident that any information I sent to my provider using the app would be received.	3.4
I felt comfortable communicating with my health care provider using the app.	2.6

T-tests were conducted in order to identify if there existed any difference of opinions among the participants groups with respect to ease of use and satisfaction (Table 5), system information and management (Table 6), and system usefulness (Table 7). No statistically significant difference of opinions was observed in relation to all sub-scales of usability factor.

**Table 5:** t-tests related to ease of use and satisfaction.

		N	Mean	Standard Deviation	*df*	*T-value*	*p-value*
Gender	Male	149	3.4	1.16	299	1.2447	.2142
	Female	152	3.6	1.59			
Age	< = 39 years	194	3.4	1.84	299	1.4880	.1378
	> 39 years	107	3.7	1.32			
Place of living	Rural	117	3.3	1.96	299	1.2245	.2217
	Urban	184	3.6	2.14			
Employment status	Employed	187	3.6	1.87	299	0.9475	.3442
	Unemployed/Retired	114	3.3	3.61			

* Statistically significant difference

**Table 6:** t-tests related to ease of system information and arrangement.

		N	Mean	Standard Deviation	*Df*	*T-value*	*p-value*
Gender	Male	149	3.3	1.08	299	1.3136	.1900
	Female	152	3.1	1.52			
Age	< = 39 years	194	3.2	1.31	299	0	1
	> 39 years	107	3.2	1.68			
Place of living	Rural	117	3.3	1.42	299	0.9519	.3419
	Urban	184	3.1	1.97			
Employment status	Employed	187	3.2	2.64	299	0	1
	Unemployed/Retired	114	3.2	1.87			

* Statistically significant difference

**Table 7:** t-tests related to ease of system usefulness.

		N	Mean	Standard Deviation	*Df*	*T-value*	*p-value*
Gender	Male	149	3.7	1.62	299	1.1574	.2480
	Female	152	3.5	1.37			
Age	< = 39 years	194	3.5	1.82	299	1.3795	.1688
	> 39 years	107	3.8	1.78			
Place of living	Rural	117	3.4	2.63	299	1.2278	.2205
	Urban	184	3.7	1.61			
Employment status	Employed	187	3.6	1.42	299	0.5158	.6064
	Unemployed/Retired	114	3.5	1.93			

* Statistically significant difference

Focusing on the findings related to the users’ intention to use the mHealth applications (as presented in Table 8), only few participants only 52% of the participants (mean rating = 2.1) stated that they would use the mHealth applications in future; while 76% participants (mean rating = 3.8) stated that they would stop using the application once the pandemic ends; and 78% participants stated that they may use the applications in future based on the necessity, but not out of interest (mean rating = 3.9).

**Table 8:** Users perceptions of intention to use.

Items	Mean Rating
I would like to use the mHealth apps in future	2.1
I would stop using the mHealth applications once the pandemic ends	3.8
I would stop/use the mHealth applications based on the necessity, but not out of interest	3.9

## Discussion & conclusion

Considering the demographic characteristics of the study populations, appropriate representation from all categories including gender, age, marital status, place of living was achieved, increasing the reliability of the data collected. Focusing on the main findings related to ease of use and satisfaction, it can be observed that only few issues such as comfortableness in using the application in social settings, amount of time required while using the app in relation to specific function, and the overall ease of use were identified. The aspect of using mHealth application in social settings can be related to the issues such as privacy and security which were identified to be the major challenges affecting the adoption of mHealth applications as identified in the previous studies [12–16]. In addition, the time taking aspect to access the functions reflects the poor or inefficient design of the applications. Accordingly, the overall ease of using the applications were rated to be average to good based on the mean rating. In relation to information management of the applications, the overall experience of using the applications were rated to be average. The issues identified were mainly related to ability to recover from the mistakes made on the application, lack of availability of all the functions in the application, and poor navigation. In consistent with these results recent studies [25–28] have identified issues related to design of the application, especially navigation, and information management. The Ministry of Health has launched various applications and improved existing applications such as Mawid, Sehha, Ashanak, Mawared etc. for various purposes including appointment booking, online consultations, medicine delivery etc. during the Covid-19 pandemic, but most of them focused on single function such as reporting of Covid-19 or acquiring permits during lock downs; and also, few with multiple purposes such as online consultations, appointment booking, medicine delivery etc. As a result of which the users had to rely on different mHealth applications for different purposes rather than using a single application that is able to cater most of the user’s healthcare needs. This could be the reason, why majority of the participants opined that they could not find the functions that they need in the applications.

In relation to the usefulness of the mHealth applications, the major issue identified was in relation to the comfort in communicating with healthcare providers. Although, the participants stated that it was convenient to communicate using the application, but they were not comfortable in communicating using the applications. This aspect may not be completely related to the issues associated with mHealth design, as it can be related to the attitudes and behavioral aspects of the mHealth users. Similar issues in the communication with healthcare providers were identified in studies related to other mHealth applications [29, 30]. Therefore, it is important that socio-cultural and behavioral aspects of the mHealth users need to be considered in designing and implementing the mHealth applications.

In a recent study conducted in Italy [31], majority of the healthcare providers used personal computers, followed by smartphones and tablets mainly for obtaining patients health-related information, to provide clinical information, and to maintain contacts with colleagues. The findings reflect positive attitudes of healthcare providers in using digital applications for obtaining patients information and for communicating with them. In addition, they are also using other applications such as social media to communicate with patients, although they are against making patients as friends, indicating that social media applications can improve communication between patients and healthcare providers in a professional dimension. Similar results were observed in another study [32], where digital devices such as wearables integrated with mHealth applications and use of social media platforms for professional communication with patients was preferred by healthcare practitioners. These studies indicate that it is also important to integrate mHealth applications with social media applications in order to facilitate improved communication and information sharing between patients and healthcare providers.

To further analyze the results, t-tests were carried out in relation to the ease of use and satisfaction (Table 5), system information and arrangement (Table 6), and usefulness (Table 7) among the different participants groups characterized by gender, age, place of living, and employment status. Analyzing the results, it can be observed that no statistically significant difference of opinions was observed in relation to all the three sub-scales of usability among the participants groups. However, interesting findings were observed in relation to the participants intention to use mHealth applications in future. Only few participants stated that they would like to use mHealth applications in future, while the majority of the participants stated that they would stop using the application once the pandemic ends or might use the applications based on the need and necessity but not out of interest. These findings can be related to the findings in [21] which highlighted that fear, anxiety and stress were the major factors during the pandemic that led to an increase in the number of mHealth users. Thus, the findings have revealed that although there are few issues associated with the usability aspects of mHealth applications, the rise in number of mHealth users can be partly linked to the necessity and emergency situations during the pandemic (due to curfews and lockdowns), and partly due to the interest and rise of awareness among the users. Considering these factors, it is necessary that the policy makers have to create awareness among the existing users in convincing them to continue using the mHealth applications as a part of digitization program (vision 2030) in Saudi Arabia.

Although interesting findings were identified in this study, there are few limitations that can be observed. Firstly, the number of participants was less than the estimated sample, because of which the generalization of results in this study must be done with care. Secondly, only survey instrument was used for collecting the data, but adopting mixed-methods approach such as both surveys and interviews could have resulted in more quality data as the behavioral and attitudinal aspects can be better captured using qualitative methods such as interviews.

However, this study has both theoretical and practical implications. The findings from this study would contribute to the healthcare literature in Saudi Arabia and also support decision-makers in making effective decisions in improving the mHealth use and adoption of eHealth technologies.
